# Transcriptional profiling identifies novel regulators of macrophage polarization

**DOI:** 10.1371/journal.pone.0208602

**Published:** 2018-12-07

**Authors:** Kimberline Y. Gerrick, Elias R. Gerrick, Anuj Gupta, Sarah J. Wheelan, Srinivasan Yegnasubramanian, Elizabeth M. Jaffee

**Affiliations:** 1 The Sidney Kimmel Comprehensive Cancer Center, The Johns Hopkins University School of Medicine, Baltimore, MD, United States of America; 2 Department of Oncology, The Johns Hopkins University School of Medicine, Baltimore, MD, United States of America; 3 The Bloomberg-Kimmel Institute for Cancer Immunotherapy, The Johns Hopkins University School of Medicine, Baltimore, MD, United States of America; 4 The Skip Viragh Center for Pancreas Cancer Clinical Research and Patient Care, The Sidney Kimmel Comprehensive Cancer Center at Johns Hopkins, Baltimore, MD, United States of America; 5 Department of Immunology and Infectious Diseases, Harvard T.H. Chan School of Public Health, Boston, MA, United States of America; Ohio State University, UNITED STATES

## Abstract

Macrophages are key inflammatory immune cells that display dynamic phenotypes and functions in response to their local microenvironment. Major advances have occurred in understanding the transcriptional, epigenetic, and functional differences in various macrophage subsets by *in vitro* modeling and gene expression and epigenetic profiling for biomarker discovery. However, there is still no standardized protocol for macrophage polarization largely due to the lack of thorough validation of macrophage phenotypes following polarization. In addition, transcriptional regulation is recognized as a major mechanism governing differential macrophage polarization programs and as such, many genes have been identified to be associated with each macrophage subset. However, the functional role of many of these genes in macrophage polarization is still unknown. Moreover, the role of other regulatory mechanisms, such as DNA methylation, in macrophage polarization remains poorly understood. Here, we employed an optimized model of human M1 and M2 macrophage polarization which we used for large-scale transcriptional and DNA methylation profiling. We were unable to demonstrate a role for DNA methylation in macrophage polarization, as no significant changes were identified. However, we observed significant changes in the transcriptomes of M1 and M2 macrophages. Additionally, we identified numerous novel differentially regulated genes involved in macrophage polarization, including *CYBB* and *DHCR7* which we show as important regulators of M1 and M2 macrophage polarization, respectively. Taken together, our improved *in vitro* human M1 and M2 macrophage model provides new understandings of the regulation of macrophage polarization and candidate macrophage biomarkers.

## Introduction

Macrophages serve critical roles as first responders in acute insults (bacterial and viral infections, and early cancerous changes) where they mediate innate inflammatory responses and influence adaptive immunity. They are extremely plastic cells that display heterogeneous phenotypes and functions depending on their environmental cues. They are often simplified into two broad polarization states that mimic the dichotomous Th1/Th2 nomenclature, termed M1 and M2 macrophages, that are the extreme opposites in terms of their phenotypes and functions. IFNγ and Toll-like receptor (TLR) agonists such as LPS are the major stimuli used to generate M1 macrophages that express inflammatory cytokines such as IL-1β, IL-12, and TNFα for intracellular pathogen and tumor cell killing [1, 2]. In contrast, M2 macrophage stimuli are more variant due to the less uniform nature of M2 macrophages themselves, as they can be further subdivided into M2a, M2b, and M2c subsets based on their functions [3, 4]. However, IL-4 and IL-13 are most commonly used to generate M2 macrophages resulting in high IL-10 and TGFβ production, which are the common denominators of all M2 macrophage subpopulations that function primarily in parasitic infections and wound healing. Due to the important roles these macrophage subsets serve in homeostatic and disease immunity, it is imperative to understand the underlying molecular and genetic differences for reliable and comprehensive biomarker identification.

*In vitro* modeling of these macrophage subsets has been extensively utilized for molecular and biomarker profiling [5–9]. Yet, these studies have limitations due to the lack of thorough validation of functional M1 and M2 macrophage models, as assessed by cytokine production. While many of these studies evaluate the expression of M1 and M2-associated cell surface markers and genes, these analyses alone are not sufficient to assess macrophage functionality. This is because, despite the fact that the dichotomous nomenclature suggests very clear distinctions in these two macrophage subsets, macrophages *in vivo* can exhibit both M1 and M2-associated cell surface marker and gene expression patterns, making it difficult to discern the exact function and impact of these cells [10–12]. As cytokine production by a particular macrophage subset is the major defining feature of its function, it is imperative to query the cytokine profile in addition to its cell surface and gene expression profile in order to accurately characterize the regulatory mechanisms of macrophage polarization.

Despite the lack of functional validation of macrophage polarization in previous transcriptional profiling studies, M1 and M2 macrophage polarization results in distinct transcriptional programs [8, 13]. Additionally, there is growing evidence that epigenetic mechanisms such as histone acetylation and methylation regulate the transcriptional differences observed in these two macrophage subsets [13–16]. While these studies provide an important foundation for the complex regulatory mechanisms governing macrophage polarization and identified novel subset biomarkers, they are still limited due to the lack of high throughput methodologies and validation of the functional consequences of newly identified genes associated with macrophage polarization, and the dearth of studies addressing the role of other regulatory mechanisms. Additionally, these studies have been predominantly described in murine macrophage polarization, which is substantially different from the human system [17].

Differences in the transcriptional programs of polarized human and murine macrophages have been identified [11, 18, 19]. Given the discrepancy between species and the lack of a comprehensively validated polarization protocol, we sought to evaluate current polarization protocols for their validity in generating human M1 and M2 macrophages. In this study, we discovered that the conventional macrophage polarization stimuli, IL-4 and IL-13, insufficiently polarized M2 macrophages at the functional level. Subsequently, we employed an optimized *in vitro* model of M1 and M2 macrophages, which we used to interrogate and correlate transcriptional changes with DNA methylation changes using RNA-Sequencing (Seq) and Methyl-CpG-Binding (MBD)-Seq. We identified many novel regulated genes that had not been previously identified; however, none of these genes were found to be regulated by DNA methylation. Because of the importance of transcriptional regulation in macrophage polarization and the need for robust markers distinguishing these two macrophage subsets, we also experimentally validated the functional roles of a subset of our novel genes. We identified cytochrome b-245 heavy chain (*CYBB*), a gene encoding a reactive oxygen species (ROS)-generating enzyme, to be important for human M1 macrophage polarization, which supports previous findings of oxidative stress as a critical regulator of macrophage function. Furthermore, we show for the first time, 7-dehydrocholesterol reductase (*DHCR7*), a gene encoding a critical enzyme in the cholesterol biosynthesis pathway, as important regulator of M2 macrophage polarization. Thus, our results offer new insights into macrophage biology and describe novel markers of macrophage polarization.

## Materials and methods

### M1 and M2 macrophage generation

Whole blood from healthy donors were obtained from the Johns Hopkins Hospital Hemapheresis and Transfusion Center, following protocols approved by the Institutional Review Board of the Johns Hopkins University and from Biological Specialty Corporation (Colmar, PA), following Food and Drug Administration approved protocols. Informed written consent was provided for each donor in accordance with the Declaration of Helsinki. PBMCs were isolated using Ficoll centrifugation, followed by CD14^+^ isolation using CD14 Microbeads (Miltenyi). Monocytes were cultured as previously described [8], with the following modifications: monocytes were plated in 6-well plates at a density of 7.0 x 10^5^ cells/cm^2^ and cultured for 6 days in RPMI supplemented with 20% fetal bovine serum (FBS) (Atlas), 1% L-glutamine, 1% sodium pyruvate, 1% non-essential amino acids, 1% penicillin streptomycin (Life Technologies), and 100 ng/mL M-CSF (Peprotech) to generate macrophages. Macrophages were cultured for an additional 72 hours in the presence of RPMI containing 5% FBS and all other reagents described above and supplemented with 20 ng/mL IFNγ (Peprotech) plus 75 ng/mL LPS (055:B5, Sigma Aldrich) for M1 polarization or 20 ng/mL IL-4 (Peprotech) plus 20 ng/mL IL-13 (Peprotech) for the first 48 hours, followed by the addition of 10 ng/mL LPS for the last 24 hours for M2 polarization.

### Flow cytometry

**Fig 1:**
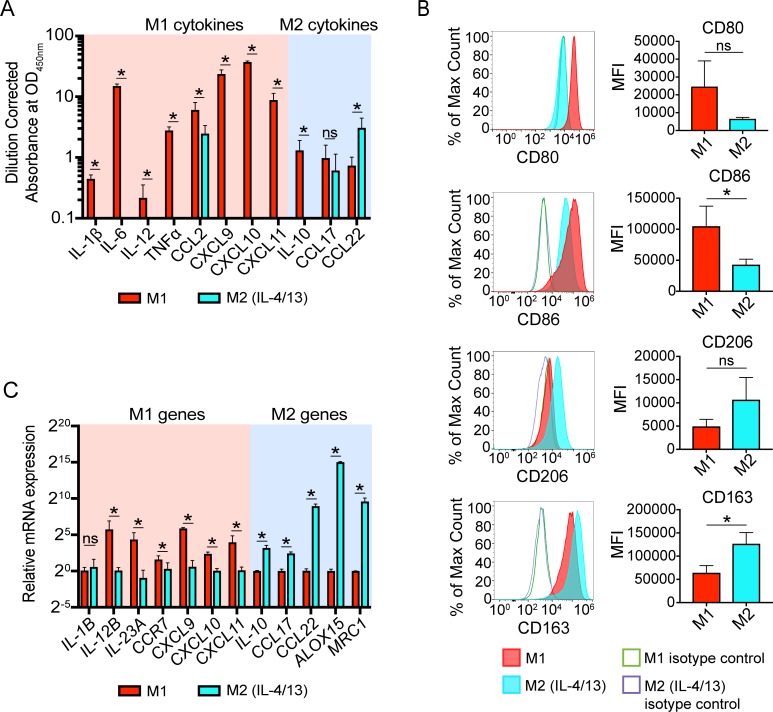
IL-4 and IL-13-polarized M2 macrophages are deficient in IL-10 and CCL17 secretion. (A) Differential cytokine secretion profiles of M1 macrophages and M2 macrophages exposed to IL-4 and IL-13. (B) Differential cell surface marker expression. MFI indicates mean fluorescence intensity. (C) Differential gene expression. For panels A and C, canonical M1 and M2 cytokines and genes are highlighted in the pink and blue background, respectively. *p<0.05 and error bars represent SEM of 3 biological replicates. ns = not significant.

**Fig 2:**
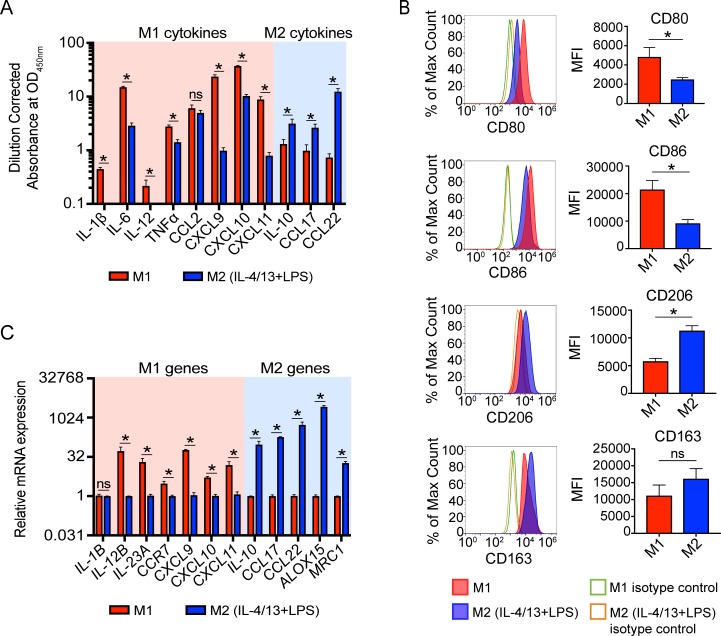
Delayed addition of LPS to conventional M2 polarization conditions phenotypically and functionally polarizes M2 macrophages. (A) Differential cytokine secretion profiles of M1 macrophages and M2 macrophages exposed to IL-4, IL-13, and LPS. (B) Differential cell surface marker expression. MFI indicates mean fluorescence intensity. (C) Differential gene expression. For panels A and C, canonical M1 and M2 cytokines and genes are highlighted in the pink and blue background, respectively. *p<0.05 and error bars represent SEM of 4 biological replicates. ns = not significant.

Macrophages were harvested using cell dissociation buffer (Invitrogen) for flow cytometric analyses. Cells were stained in LiveDead Aqua (Invitrogen) and then incubated with human FcR blocking reagent (Miltenyi). For purity analyses post CD14^+^ isolation, cells were stained with anti-CD14 FITC clone TÜK4 (Miltenyi) and anti-CD3 PE clone REA613 (Miltenyi). For M1 and M2 macrophage phenotypic analyses, cells were stained with anti-CD206 FITC clone DCN228 (Miltenyi), anti-CD163 PEDazzle clone GHI/61 (BioLegend), anti-CD80 BV421 clone 2D10 (BioLegend), and anti-CD86 AF700 clone FUN-1 (BD Pharmingen). Isotype controls were also stained in parallel. Data for Figs 1 and 2 were collected on a Gallios flow cytometer (Beckman Coulter) and subsequent data on a Cytoflex flow cytometer (Beckman Coulter). FlowJo software version 10.4.2 (Tree Star) was used to analyze the results. Cells were gated to exclude dead cells before differential expression analyses.

### RNA extractions and RT-qPCR

RNA was extracted using the RNeasy Mini kit (Qiagen), genomic DNA digested with TURBO DNase (Ambion), and RNA purified using spin columns (Qiagen). cDNA synthesis was performed using the Superscript VILO cDNA synthesis kit (Invitrogen). Real-time quantitative PCR was performed using Taqman Gene Expression Assays (Applied Biosystems) on a StepOnePlus Real Time PCR System thermal cycler (Applied Biosystems) and analyzed by the StepOne software V2.1. Transcript levels were quantified using the ΔΔCt method and normalized to the housekeeping gene *TBP*. The expression levels of canonical M1 genes are reported relative to M2 macrophages and conversely, the expression levels of canonical M2 genes are reported relative to M1 macrophages.

### RNA-sequencing and differential gene expression analysis

RNA from 4 biological replicates were used for RNA-sequencing, which was done by the Sidney Kimmel Comprehensive Cancer Center (SKCCC) Next Generation Sequencing (NGS) Core. Libraries were prepared using the TrueSeq Stranded Total RNA kit (Illumina) and sequenced on HS2500 Rapid Run instrument (Illumina). RSEM-1.2.29 was used to align raw sequencing files to hg19 reference genome and to calculate transcript expression levels, which were then used for differential gene expression analysis using DESeq2 package [20].

### DNA extractions and MBD-sequencing

Genomic DNA from the same biological replicates used for RNA-Seq was extracted using DNeasy Blood and Tissue Kit (Qiagen) and submitted for MBD-sequencing, which was also done by the SKCCC NGS Core (PMID: 28733453). Sonicated genomic DNA (target modal size 200–500 bp) was enriched for methylated DNA using the EpiMark Methylated DNA Enrichment Kit (New England Biolabs) according to manufacturer’s protocol. A matched unenriched total input fraction was also analyzed for each sample. The enriched methylated and total input fractions were then subjected to NGS library preparation using the Thruplex library preparation kit (Rubicon). Alignments were performed with bowtie 2.2.5 and duplicated alignments were removed by samtools 0.1.19.

### Methylation peak identification and differential enrichment analysis

Bedtools was used to compute the per-base genome coverage. Methylation peaks were identified using methylation peak Seeker (methSeek) a modified version of a bacterial small RNA search tool [21]. Briefly, methSeek scans along the genome within transcript promoters to identify regions with coverage above a defined threshold as methylation peaks. The parameters were as follows: promoters were defined as 5000 bp upstream and 2000 bp downstream a transcriptional start site, the 5’ end of a methylation peak had a read depth greater than or equal to 10 and the 3’end had a read depth less than 10. methSeek was used to identify methylation peaks in M1 and M2 samples from 4 biological replicates, which was then compiled into a master list. To condense the master list, methylation sites with start and stop coordinates within 100 bp of each other were merged as one methylation site with the new start and stop coordinates set to more upstream 5’ and downstream 3’ coordinates. Bedtools was used to compute the coverage of each methylation site for all samples. DESeq2 package was then used for differential methylation enrichment analysis.

### ELISA

Supernatant from macrophage cultures were harvested and measured for IL-1β, IL-6, IL-12, TNFα, CCL2, CXCL9, CXCL10, CXCL11, IL-10, CCL17, and CCL22 expression using custom multi-analyte ELISArray kits (Qiagen), according to the manufacturer’s instructions. Samples were diluted to stay within the dynamic range of the assay and the dilution factors were accounted for when calculating the corrected absorbance values. Absorbance was measured using a microplate reader (Molecular Devices).

### siRNA transfection

siRNA-mediated gene knockdown was performed as described [22], with the following modifications: monocytes were cultured as described above for 6 days. The next day, cells were incubated in the presence of 0.4 mL RPMI containing 5% FBS, 1% L-glutamine, 1% sodium pyruvate, and 1% non-essential amino acids. Per well, cells were transfected with 50 nM ON-TARGETplus SMARTpool siRNAs (Dharmacon) and 24 µL HiPerfect transfection reagent (Qiagen) diluted in 0.4 mL Opti-MEM medium for six hours. Six hours after transfection, cells were supplemented with RPMI medium described above and 100 ng/mL M-CSF overnight. The following day, cell culture medium was refreshed and macrophages were polarized to generate M1 or M2 macrophages using the conditions described in the previous section. In transfections knocking down M1 macrophage-associated genes, macrophages were polarized to M1. Conversely, in transfections knocking down M2 macrophage-associated genes, macrophages were polarized to M2. Following polarization, RNA, cells, and supernatant were collected for RT-qPCR, flow cytometry, and ELISA, respectively. Cells were transfected with ON-TARGETplus SMARTpool non-targeting controls (designated siControl) as negative controls and siGLO red transfection indicator to determine transfection efficiency.

### Gene set enrichment analysis (GSEA)

The top differentially expressed gene list ranked by greatest log2 fold change was analyzed using the Broad Institute’s GSEA software [23]. Significantly enriched gene sets were identified using the FDR < 0.01 cutoff.

### Statistical analyses

GraphPad Prism version 7.0 software was used for all statistical analyses. Data are presented as mean ± SEM of at least 3 independent experiments. Student t-test was used to assess differences between groups. Statistical significance was defined as p-value < 0.05.

## Results

### LPS rescues IL-10 and CCL17- deficiencies in IL-4 and IL-13-polarized M2 macrophages

In order to study the underlying biological differences in human macrophage subsets, we utilized an *in vitro* model that simplified these subsets into two polarized states, termed M1 and M2 macrophages. Conventional protocols use IFNγ and LPS to generate M1 macrophages and IL-4 and IL-13 to generate M2 macrophages [1, 3, 24]. In the process of validating previously published protocols for M1 and M2 macrophage polarization, we found that while the traditional polarization conditions produced M1 macrophages with expected cytokine, cell surface, and gene expression profiles (Fig 1) and M2 macrophages with expected cell surface and gene expression profiles (Fig 1B and 1C), M2 macrophages lacked enhanced secretion of the hallmark M2 cytokines IL-10 and CCL17 when compared to M1 macrophages (Fig 1A). Notably, there was a complete absence of IL-10 secretion from M2 macrophages, which was surprising as IL-10 is a major cytokine for M2 function [25–27]. Because LPS is known to be a major inducer of IL-10 production in macrophages [28–30], and while cytokine exposure can be separated *in vitro*, it is unlikely that these cytokines are entirely separated within the tissue microenvironment. Thus, we hypothesized that the addition of LPS to M2 polarization conditions would rescue this deficiency. To this end, we found that LPS exposure following initial culture with IL-4 and IL-13 rescued the IL-10 deficiency observed with the standard polarization protocol (Fig 2A), while maintaining other M2 macrophage characteristics (Fig 2B and 2C). This is concurrent with a previous study that observed restored IL-10 production from IL-4-primed macrophages upon LPS stimulation [31]. Additionally, CCL17 and CCL22, other canonical M2 cytokines, were further upregulated in these conditions, indicating an important role for LPS exposure in M2 polarization and not just regulation of IL-10 in this model (Fig 2A).

### DNA methylation is not the primary mechanism regulating macrophage polarization

**Fig 3:**
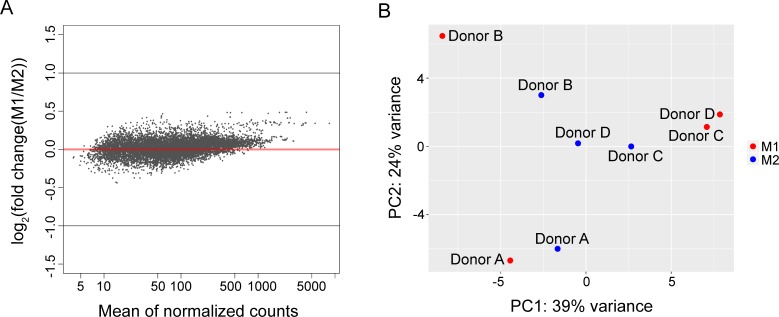
DNA methylation is not a major regulatory mechanism of macrophage polarization. (A) MA plot (B) PCA plot of the promoter DNA methylation sites identified by methSeek in M1 and M2 macrophages. Red and blue data points represent M1 and M2 macrophages, respectively.

We used the optimized protocol (designated M2 in subsequent figures) that generated phenotypic and functional M1 and M2 macrophages to further query the regulatory mechanisms underlying biological differences in M1 and M2 macrophages. While there is evidence that transcriptional regulation is a key regulatory mechanism of macrophage polarization [5–9], there is a paucity of information on the role of DNA methylation in regulating macrophage polarization. To interrogate global DNA methylation changes between these two macrophage subsets, we performed MBD-Seq and developed a bioinformatic methylation peak finder tool, methylation peak Seeker (methSeek), that identified DNA methylation peaks using a read depth-centric approach. methSeek utilizes a sliding window approach to define the 5’ and 3’ ends of methylation peaks that meet user-defined coverage thresholds. We first interrogated methylation peaks in promoters, since promoter methylation is the most well documented location of regulatory DNA methylation sites [32–37]. Of the methylation peaks identified by methSeek in all donor samples, none were significantly differentially enriched (defined by |log_2_FC(M1/M2)| > 1.0) between M1 and M2 macrophages (Fig 3A). We also interrogated intragenic methylation peaks, none of which were significantly differentially enriched between M1 and M2 samples (S1 Table). Interestingly, sample variance analysis using Principal Component Analysis (PCA) showed that the samples were more similar based on donor origin than on macrophage polarization status. (Fig 3B). This suggests that macrophage polarization is not primarily regulated by DNA methylation. Furthermore, the observed differences in DNA methylation are predominantly due to inherent biological differences among individuals and not differential macrophage polarization. Thus, we concluded that DNA methylation may not be regulating differences in M1 and M2 macrophages. This led us to investigate whether differential transcriptional programming alone is a major regulator.

### Transcriptome analysis reveals novel genes associated with functional M1 and M2 macrophages

**Fig 4:**
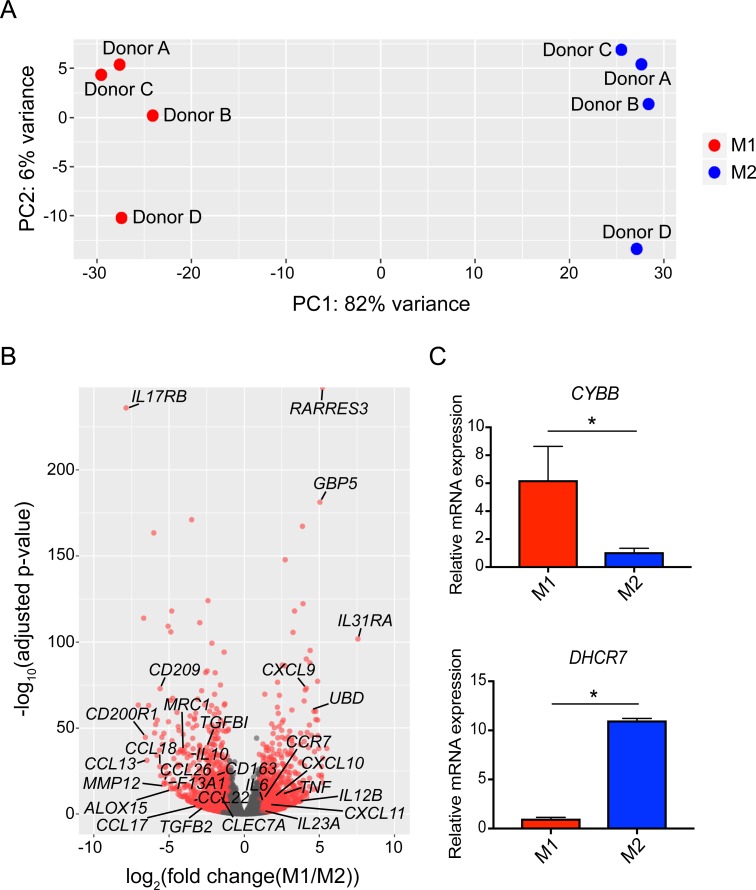
Transcriptional profiling reveals transcriptional regulation of macrophage polarization and novel markers of M1 and M2 macrophages. (A) PCA plot of all genes interrogated by RNA-Seq. Red and blue data points represent M1 and M2 macrophages, respectively. (B) Volcano plot. Red data points represent significantly differentially expressed genes (adjusted p-value < 0.05 and |log_2_FC(M1/M2)| > 1.0). Canonical M1 and M2 genes are labeled. (C) RT-qPCR validation of *CYBB* and *DHCR7* gene expression in M1 and M2 macrophages. *p<0.05 and error bars represent SEM of 3 biological replicates.

Although differential transcriptional profiles in M1 and M2 macrophages have been well-documented, these studies were performed based on the conventional IL-4 and IL-13-polarization conditions to generate M2 macrophages. Because we generated phenotypic and functional M2 macrophages to interrogate transcriptional differences in M1 and M2 macrophages, we hypothesized that our RNA-Seq dataset would yield novel markers to distinguish these two macrophage subsets. We analyzed sample variance in the RNA-Seq dataset using PCA, which revealed that 82% of the variance in all samples was due to different macrophage polarization states and not donor origin (Fig 4A). This indicates that opposing macrophage polarization conditions results in very distinct transcriptomes that regulate macrophage polarization. To define the top differentially expressed genes, we selected for genes with an adjusted p-value < 0.05 and |log_2_FC(M1/M2)| > 1.0. Of the 26,341 genes interrogated in RNA-Seq, 2,200 genes met these criteria (Fig 4B, red data points, S2 Table). Many of these genes are previously reported canonical genes of M1 and M2 macrophages (Fig 4B), which confirmed the validity of our optimized macrophage polarization protocol. However, 1,826 of the 2,200 top differentially expressed genes are not known canonical genes of macrophage polarization, when compared to previously published datasets [6, 8, 9]. Thus, we hypothesized that these genes might have important and novel roles in macrophage polarization.

### *CYBB* and *DHCR7* regulate macrophage polarization

**Fig 5:**
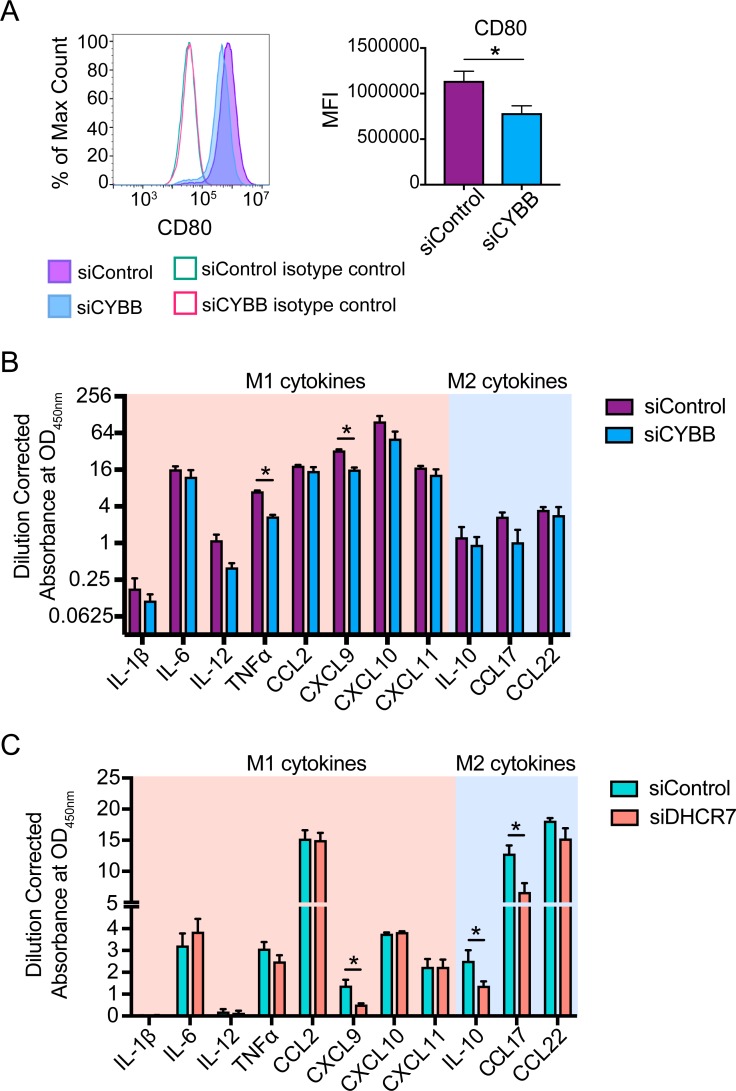
*CYBB* and *DHCR7* regulate M1 and M2 macrophages. (A) Differential surface CD80 expression of M1 macrophages exposed to either siControl or siCYBB. MFI indicates mean fluorescence intensity. Differential cytokine secretion profile of (B) M1 macrophages exposed to siControl or siCYBB (C) M2 macrophages exposed to siControl or siDHCR7. Canonical M1 and M2 cytokines are highlighted in the pink and blue background, respectively. *p<0.05 and error bars represent SEM of at least 3 biological replicates.

To dissect which of these genes had a functional consequence on macrophage polarization, we selected a set of genes that were highly differential in our dataset but not previously described as canonical markers of macrophage polarization. After applying an abundance cutoff, genes were prioritized based on differential expression, and the top 14 genes (Table 1), which we confirmed by RT-qPCR (Fig 4C and S1 Fig, designated untransfected M1 and M2), were selected for further analyses. Of these genes, 4 have been previously associated with differential expression in M1 and M2 macrophages, whereas 10 are novel as of this study [5–9]. However, few of these genes have been shown to have an effect on human macrophage polarization. Subsequently, we performed siRNA-mediated gene knockdown of each of these genes (S1 Fig) and tested whether knockdown of these genes during polarization conditions resulted in alterations in canonical M1 and M2 cell surface and cytokine expression profiles. As a proof-of-principle experiment, we first knocked down cytochrome b-245 beta chain (*CYBB*), an M1 macrophage-associated gene that encodes the catalytic subunit of NADPH oxidase responsible for generating reactive oxygen species (ROS), in M1 macrophages. We selected *CYBB* as our model gene because the protein product, NADPH oxidase, has been shown to be important for the inflammatory functions of M1 macrophages derived from both murine and human models [38–43]. Knockdown of *CYBB* in M1 macrophages resulted in significant downregulation of CD80 cell surface expression and TNFα and CXCL9 secretion using our panel of canonical markers of macrophage polarization (Fig 5A and 5B). This shows that *CYBB* expression promotes M1 macrophage polarization and its effects are abrogated upon gene knockdown, resulting in dampened M1 characteristics that are reminiscent of M2 macrophages. These results are consistent with previous studies showing the critical role of various NADPH oxidase subunits including the *CYBB* gene product and their superoxide and ROS products in the inflammatory and cytotoxic functions of M1 macrophages [38–43]. Furthermore, this highlights oxidative stress as an important and conserved regulator of macrophage polarization across multiple species.

**Table 1:** Genes selected for siRNA-mediated gene knockdown.

Gene Name	Gene Symbol	Log2FoldChange(M1/M2)	Adjusted p-value
Clusterin	*CLU*	4.8831722	6.95E-78
Phospholipase A2 Group XVI	*PLA2G16*	4.16649866	2.64E-13
ATP Binding Cassette Subfamily G Member 1	*ABCG1*	3.65030094	5.92E-09
Basic Helix-Loop-Helix Family Member E41	*BHLHE41*	2.68190157	2.02E-26
BCL2 Like 14	*BCL2L14*	2.457426634	5.72E-35
Cytochrome b-245	*CYBB*	2.350189727	3.24E-41
Integrin Beta Chain Beta 3	*ITGB3*	-4.8025146	2.06E-43
Serpin Family B Member 4	*SERPINB4*	-4.2730023	2.59E-11
A-Kinase Anchoring Protein 5	*AKAP5*	-3.795068132	1.41E-33
7-Dehydrocholesterol Reductase	*DHCR7*	-3.593065496	6.84E-29
Sestrin 3	*SESN3*	-3.541094785	8.89E-35
Interferon Induced Protein with Tetratricopeptide Repeats 1	*IFIT1*	-3.426918128	8.79E-45
Alpha-2-Macroglobulin	*A2M*	-3.372507505	4.19E-22
Fatty Acid Desaturase 2	*FADS2*	-3.091196156	1.49E-11

The Log2FoldChange(M1/M2) and adjusted p-values were calculated using the DESeq2 analysis package.

Accordingly, we knocked down the other 13 genes to determine their role in macrophage polarization. Upon siRNA-mediated knockdown of 12 of these genes, we did not observe differences in the expression of canonical cell surface markers or cytokines (S2 Fig). This indicates that while 12 of these 13 differentially expressed genes are possibly important in and associated with macrophage function, they do not directly regulate previously identified markers associated with M1 and M2 polarization.

Interestingly, we identified 7-dehydrocholesterol reductase (*DHCR7)*, an M2 macrophage-associated gene that encodes an enzyme that catalyzes the final step of cholesterol biosynthesis, to be important for M2 macrophage polarization. Upon *DHCR7* gene knockdown, M2 macrophages did not display significant changes in their cell surface marker expression but displayed significant reduction of IL-10 and TARC secretion (Fig 5C), providing a role for *DHCR7* in regulating M2 macrophage functionality. These data therefore support a role for the cholesterol pathway in macrophage polarization. This notion was supported by gene set enrichment analysis, which demonstrated that the cholesterol homeostasis pathway was significantly upregulated in M2 macrophages (S3 Fig) [23]. Moreover, certain cholesterol intermediates and derivatives have been shown to promote M2 macrophage phenotypes by indirectly suppressing transcription of pro-inflammatory transcription factors, NFkB and AP-1, via liver X receptors (LXRs) activation [44–46]. Taken together, these data implicate *CYBB* and *DHCR7* as key regulators of M1 and M2 macrophage polarization, respectively.

## Discussion

Much of our understanding of the regulation of human macrophage subsets comes from *in vitro* models of M1 and M2 macrophages. However, there is still an incomplete characterization of these macrophage subsets and their regulatory mechanisms. Here, we developed an optimized model of M1 and M2 macrophages to evaluate the role of DNA methylation in their differential transcriptomes using high throughput approaches. Although we did not find a significant role of DNA methylation in macrophage polarization, we found significantly distinct transcriptional changes that generated a list of genes that had not been previously associated with macrophage polarization. These results provide a more comprehensive model of human macrophage subsets, a more global understanding of epigenetic and transcriptional regulation of macrophage polarization, as well as the characterization of critical regulators of M1 and M2 macrophages. Importantly, these findings provide valuable insights into macrophage activation and macrophage-directed therapeutic strategies.

By interrogating macrophage cytokine production, we validated that the established M2 macrophage stimuli, IL-4 and IL-13, did not sufficiently functionally polarize M2 macrophages; the addition of LPS was necessary to generate fully phenotypic and functional M2 macrophages. This highlights the importance of functional validation in combination with phenotypic validation, given the complex and heterogeneous nature of macrophages. While the use of LPS to generate M2 macrophage subsets is not novel, its use in the context of IL-4 and IL-13-primed macrophages is not widely used. Importantly, our results provide evidence that the macrophage polarization methodologies we used should be ubiquitously employed for studies involving macrophage polarization. Furthermore, these results have larger implications for macrophage polarization. Despite being a canonical stimulus for M1 macrophages, the fact that LPS is essential for M2 macrophage production of IL-10 and CCL17 in our model suggests that the functional response of macrophages to LPS and other stimuli are indeed context-dependent. Additionally, these findings suggest that these macrophage subsets are likely not confined to discrete compartments in physiological and pathological settings. They are likely exposed to various and perhaps opposing cytokines that coordinately influence their function. Thus, while the dichotomous nomenclature of macrophage polarization is useful for mechanistic studies, it still has its limitations in depicting the pleiotropic nature of macrophages. Therefore, it is important to note the spectrum model of macrophage activation, where M1 and M2 macrophages are at the opposite ends and macrophages with overlapping M1 and M2 features lie in the middle of this spectrum. Indeed, in our studies, knock down of *CYBB* in M1 macrophages and *DHCR7* in M2 macrophages did not fully polarize one macrophage subset into the other, rather it resulted in a macrophage with characteristics of both macrophage subsets. Future studies pairing *in vitro* macrophage models with *in vivo* macrophages would be extremely informative for understanding the full range of macrophage activation states.

Using our optimized protocol for macrophage polarization, we found no evidence of DNA methylation regulating macrophage polarization; however, this finding may be a result of the nature of MBD-Seq, as its sensitivity is enhanced for regions with a high density of methylated CpG, such as CpG islands [47–50]. Perhaps differences in DNA methylation sporadically across the genome are present, but may not have been captured at the single nucleotide level by MBD-Seq. Nevertheless, subtle changes in DNA methylation may still be important for macrophage polarization, as they can work concomitantly with histone modifications to remodel the nucleosome for coordinated transcriptional regulation [15, 51–55]. While current studies of the epigenetics of macrophage polarization focus mainly on histone acetylation and methylation in murine macrophages [14, 56–66], further elucidation of the role of DNA methylation in human macrophage polarization would provide a deeper understanding of the complete epigenetic landscape in M1 and M2 macrophages.

Our findings also provide new insights into effective epigenetic therapies directed at reprogramming macrophage functions. In recent years, two major classes of epigenetic drugs, histone deacetylase (HDAC) and DNA methyltransferase (DNMT) inhibitors, have demonstrated significant potential as immunomodulatory agents in diseases such as cancer [67–69]. Despite this, the lack of significant differences in DNA methylation between M1 and M2 macrophages in our dataset and the fact that DNMT inhibitor activity depends on their integration into the genome of highly proliferative cells such as cancer cells [70, 71] and not in less proliferative macrophages [72–75], suggest that DNMT inhibitors may not be the most effective epigenetic drug for macrophage modulation. However, it is important to recognize that DNMT inhibitors can still indirectly affect macrophage function by directly modulating cancer cells and other immune cells in the local microenvironment. Alternatively, HDAC inhibitors may serve as more promising epigenetic modifiers, as their effects are not limited to highly proliferative cells. Indeed, HDAC inhibitors such as vorinostat and trichostatin A have been shown to alter macrophage gene expression in response to LPS [56, 76, 77], highlighting their utility as macrophage reprogramming agents.

We also identified *CYBB* and *DHCR7* as regulators of macrophage polarization. Previous studies have shown the importance of *CYBB* in the bactericidal and inflammatory activity of macrophages, due to the role of the protein product, NADPH oxidase, in TLR- and IFNγ-dependent oxidative burst and pro-inflammatory cytokine synthesis [38–41]. These functions are hallmark features of M1 macrophages, and therefore are highly consistent with our results showing the increased expression and functional significance of *CYBB* in M1 macrophage polarization. Macrophage redox status strongly influences its phenotype and function and has been studied extensively in inflammatory disorders such as atherosclerosis, chronic granulomatous disease, and cancer, where macrophage redox imbalance exacerbates disease progression. Accordingly, this implies the exciting potential of targeting *CYBB* in addition to other members of the ROS signaling pathway for macrophage reprogramming and immunomodulatory therapies.

While *DHCR7* had been previously identified as an M2-associated gene in a published microarray-based screen using the traditional M2 polarization condition [9], this is the first time that *DHCR7* has been experimentally validated to be important for M2 macrophage polarization. The exact mechanism of *DHCR7*-mediated regulation of M2 macrophage polarization remains unclear. Interestingly, a previous study demonstrated that desmosterol accumulation upon inhibition of DHCR24, another terminal enzyme in the cholesterol biosynthesis pathway, in macrophage foam cells directly suppressed inflammatory-response genes [45]. Although DHCR24 and DHCR7 both synthesize cholesterol, their substrates, desmosterol and 7-dehydrocholesterol (7DHC), respectively, have very distinct properties and functions [78–80]. As such, one possible explanation for our findings is that the accumulation of 7DHC upon *DHCR7* inhibition may interact differently with inflammatory mediators to induce the expression of inflammatory genes. Future studies will be necessary to determine how DHCR7 and its substrate 7DHC interact with inflammatory mediators and if other members of the cholesterol biosynthesis pathway are also involved. Given the need for additional robust markers of macrophage polarization, it will also be important to evaluate the utility of *DHCR7* as a marker to distinguish M2 macrophages from M1 macrophages *in vivo* and as a target of macrophage repolarization therapies. Furthermore, these results are particularly exciting because they uncover the cholesterol pathway as another potential target for macrophage reprogramming therapies. The role of cholesterol homeostasis in macrophage immunity has been extensively studied in atherosclerosis, where cholesterol activates pro-inflammatory signaling pathways via engagement with macrophage pattern recognition receptors such as TLRs or engulfment by macrophages to accumulate cellular cholesterol [46]. One way to modulate macrophage function is through the use of statins, which are used to treat atherosclerosis by reducing plasma cholesterol levels. In fact, several statins have been shown to inhibit macrophage secretion of MMP-9 [81] and expression of IL-6 and TNFα in response to LPS [82, 83]. It would be of great interest to determine if the macrophage modulatory activity of statins in atherosclerosis could also be observed in other inflammatory diseases such as cancer and autoimmunity.

In addition to *CYBB* and *DHCR7*, we also knocked down 16 other genes in our siRNA screen but did not observe obvious phenotypic and functional changes at the protein level. However, it is important to note that protein changes may still be occurring given that our analyses were not exhaustive. It is likely that these genes do not regulate certain markers of macrophage polarization but are still important for macrophage function. This highlights the need for a more comprehensive panel of markers of macrophage polarization for improved phenotypic and functional characterization. Nevertheless, the subtle protein alterations observed may also be due to the fact that these genes are not the master regulators of their pathways or that they are part of a redundant pathway in which multiple genes in a given pathway need to be knocked down to observe significant changes. How these genes function in macrophage polarization and whether these genes can be used as markers of different macrophage subsets remain to be elucidated. Finally, there is a plethora of other novel genes in our dataset that remain to be explored for their biomarker potential.

## Supporting information

S1 Table List of the methylation peaks identified by methSeek. Methylation peaks identified by methSeek in promoter and intragenic regions are listed.
(TXT)

S2 Table Top differentially expressed genes from RNA-Seq dataset. These genes were selected based on the following criteria: adjusted p-value < 0.05 and |log_2_FC(M1/M2)| > 1.0. Genes are sorted based on decreasing log2(fold change(M1/M2)).
(TXT)

S1 Fig RT-qPCR validation of differential expression and knockdown levels of the genes selected for siRNA knockdowns. Expression levels of (A) M1-associated genes in untransfected M1 and M2 macrophages (red and blue bars, respectively) and M1 macrophages exposed to siControl and siRNAs (purple and light blue bars, respectively). (B) M2-associated genes in untransfected M1 and M2 macrophages (red and blue bars, respectively) and M2 macrophages exposed to siControl and siRNAs (turquoise and coral bars, respectively). *p<0.05 and error bars represent SEM of 3 biological replicates
(EPS)

S2 Fig Knockdown of 12 genes does not significantly alter the expression of a subset canonical cell surface markers and cytokines associated with macrophage polarization. Differential cell surface marker expression of (A) M1 macrophages exposed to siRNA (B) M2 macrophages exposed to siRNA. MFI indicates mean fluorescence intensity. Differential cytokine secretion profiles of (C) M1 macrophages exposed to siRNA (D) M2 macrophages exposed to siRNA. Canonical M1 and M2 markers and cytokines are highlighted in the pink and blue background, respectively. *p<0.05 and error bars represent SEM of at least 3 biological replicates.
(EPS)

S3 Fig The cholesterol homeostasis pathway is significantly upregulated in M2 macrophages. Gene set enrichment analysis (GSEA) enrichment plot showing the significant enrichment of the cholesterol homeostasis gene set in M2 macrophages compared to M1 macrophages (FDR < 0.01) (left panel). Heatmap of the significantly differentially expressed genes that were enriched in the cholesterol homeostasis gene set (right panel).
(EPS)
